# Patient Care of Pregnant Women With Chronic Myeloid Leukemia in a Resource Limited Setting—Case Reports From Ghana

**DOI:** 10.1155/crh/8111388

**Published:** 2026-08-29

**Authors:** Amma Benneh-Akwasi Kuma, Leticia Lokko Hanson, Nii Boi-Doku Pepra-Ameyaw, George Dennis Obeng, Marcell Csanádi

**Affiliations:** ^1^ Korle-Bu Teaching Hospital, Accra, Ghana, kbth.gov.gh; ^2^ University of Ghana Medical School, Accra, Ghana, ug.edu.gh; ^3^ Syreon Research Africa, Accra, Ghana

## Abstract

Although life expectancy and survival have improved for CML patients due to the advent of tyrosine kinase inhibitor (TKI) therapies, the quality of life and family planning for women still hold challenges, especially in a resource‐limited setting where routine molecular monitoring and pregnancy‐compatible therapeutic options are limited. Due to their teratogenicity, TKI therapies are generally considered to be contraindicated during pregnancy. Here, we present case reports of three patients from the Korle‐Bu Teaching Hospital in Accra, Ghana, who were diagnosed with CML prior to pregnancy. A 29‐year‐old, 19‐year‐old and 33‐year‐old female were diagnosed with CML at the clinic. All patients initiated imatinib, which was stopped after confirming pregnancy. Patients were subsequently monitored with a monthly full blood count (FBC). While one of the patients was lost to follow‐up, the other two patients delivered without adverse events or fetal abnormalities and were able to restart imatinib therapy within 6 months. Based on the presented cases, a pragmatic framework emerges: (1) embed comprehensive counselling at diagnosis; (2) encourage planned conception only after sustained molecular remission; (3) stop TKIs upon pregnancy confirmation; (4) monitor with clear, communicated thresholds for intervention; (5) introduce cytoreduction after the first trimester only when clinically indicated and consent given; and (6) align postpartum feeding plans with timely TKI re‐initiation. As a conclusion, in contexts where social pressure to bear children is strong and pregnancy‐safe treatments are limited, transparent counselling, a remission‐first strategy, and tightly coordinated hematology–obstetric care offer the best path to balancing reproductive autonomy with maternal and fetal safety.

## 1. Introduction

Chronic myeloid leukemia (CML) is a myeloproliferative neoplasm characterized by the uncontrolled growth of myeloid cells at different stages of maturation. CML accounts for about 15% of adult leukemias. In Western countries, the median age at presentation is between 50 and 60 years, with 12% to 30% of patients at diagnosis being older than 60 years [1]. The median age at diagnosis is typically lower in African and Asian countries [2]. Patients may present in three disease phases: chronic phase, accelerated phase, and blast phase (blast crisis). The latter is characterized by an increasing percentage of blasts of the myeloid, lymphoid, or mixed/undifferentiated lineage [3]. Patients also become more resistant to treatment in each successive phase. Due to the advent of tyrosine kinase inhibitor (TKI) therapies, patients in the chronic phase can now have a life expectancy comparable to that of the general population. However, most patients with CML require lifelong TKI therapy [4].

Although survival rates have significantly improved, the quality of life and family planning for women still hold challenges. Due to their potential teratogenicity, TKI therapies are generally considered to be contraindicated during pregnancy. Patients who wish to become pregnant and meet standardized treatment‐free remission (TFR) criteria can stop the therapy. However, in many cases, this decision becomes less obvious. Examples include an unplanned pregnancy or when pregnant women are diagnosed with CML. There remains a paucity of reliable evidence available to guide treatment in such cases [5]. Former studies reported that disease management requires not only the coordination of drug therapy and fetal monitoring but also close communication between multiple medical disciplines [6].

Here, we present case reports of three patients from the Korle‐Bu Teaching Hospital in Accra, Ghana, who were diagnosed with CML prior to pregnancy. Informed consent was obtained from all patients and was documented in their medical records.

Korle‐Bu Teaching Hospital is the largest tertiary hospital in Ghana. Referrals are received from all 16 political regions of Ghana as well as other countries such as Sierra Leone, Liberia, Togo, and the Ivory Coast. At the Department of Hematology, currently there are 4 consultant hematologists, 9 specialists, and 11 residents. There are approximately 15–20 newly diagnosed CML cases annually with a median age of 40 years. Healthcare costs are mostly paid by the patients out of pocket. However, TKIs are available through donations from Max Access Solutions, which significantly reduces the financial burden for patients. As a result, currently, there are over 200 patients on TKIs. The first‐line therapy is imatinib, and the therapies available for second‐line are nilotinib, bosutinib, and ponatinib.

As part of the institutional protocol, pregnancy is thoroughly discussed at diagnosis and before treatment. Contraception is emphasized among the women. Despite the counselling sessions, pregnancies still occur, and thus it becomes a journey embarked on by physicians and patients to ensure the survival of both mother and baby. In the past 11 years, a total of 6 women diagnosed with CML have become pregnant; 1 woman opted to end the pregnancy, and 5 carried the pregnancy to term and delivered. Of these 5 patients, three were followed through pregnancy, while two were lost to follow‐up shortly after reporting pregnancy.

According to the protocol, TKI treatment is stopped upon confirmation of pregnancy, and monthly full blood count (FBC) monitoring is done. Other tests such as BCR::ABL1 are generally not accessible for regularly monitoring patients’ conditions, as patients are required to pay for them out of pocket. When the clinical condition requires treatment for CML, hydroxyurea is initiated. The conditions to start the therapy include a total white blood count (WBC) ≥ 100 × 10^9^/L and a platelet count ≥ 600 × 10^9^/L. Hydroxyurea can be initiated during the second trimester after consent is given by the patient. After delivery, the TKI treatment is promptly re‐started after breastfeeding is discontinued. Interferon therapy is currently not available in the country.

## 2. Case Presentations

Patient one was a 29‐year‐old female diagnosed in 2015 following a 10‐month history of an abdominal mass. Significant findings on examination were anemia and massive splenomegaly (20 cm below the subcostal margin). Diagnosis was made by bone marrow aspirate and RT‐PCR (transcript level 84%). Initial FBC was Hb–9.4 g/dL; WBC–210.13 × 10^9^/L; platelet count–617 × 10^9^/L. Her EUTOS long‐term survival (ELTS) score (1.9186) was consistent with intermediate‐risk disease. The patient was started on imatinib 400 mg daily in May 2015 and attained a complete hematologic response. WBCs remained within normal ranges during the period of therapy. The patient presented about two years later with a two‐month history of amenorrhea. After a positive pregnancy test, the patient was enrolled in the antenatal clinic. Imatinib treatment was withheld, and monthly FBC monitoring was done. WBC remained relatively stable until 7 months into pregnancy (Table 1).

**TABLE 1:** FBC results of patient one during pregnancy.

	05/2017	06/2017	07/2017	08/2017	09/2017	10/2017	11/2017^∗^
HB (g/dL)	11.6	11.3	11.2	12.0	11.2	11.8	11.7
WBC (x 10^9^/L)	4.04	7.33	7.94	10.53	15.95	20.4	16.22
PLT (x 10^9^/L)	173	260	266	239	290	257^∗∗^	276^∗∗^

^∗^FBC done during the month of delivery.

^∗∗^tip of spleen palpable.

The delivery was by spontaneous vaginal delivery. There were no adverse events or fetal abnormalities. The Apgar score at 1 min was 8/10 and 10/10 at 5 min. After delivery, the WBC of the mother started to rise gradually. The 2 months postpartum FBC was Hb–14.9 g/dL; WBC–28.5 × 10^9^/L; platelet count–475 × 10^9^/L. The 4 months postpartum FBC was Hb–12.8 g/dL; WBC–36.4 × 10^9^/L; platelet count–500 × 10^9^/L. Imatinib therapy was restarted in April 2018 after the patient stopped breastfeeding. She has since achieved and maintained a complete hematologic response on imatinib 400 mg daily. FBC results have been stable since therapy was restarted. Results from the last visit: Hb–12.4 g/dL; WBC–3.65 × 10^9^/L; platelet count–307 × 10^9^/L. Her child is also in good health.

Patient two was a 19‐year‐old female diagnosed in 2022. The patient had a 9‐month history of abdominal distention. On presentation, she was noticed to be mildly pale and had a tinge of jaundice. Both liver and spleen were 18 cm palpable below their respective subcostal margins. Other systems were essentially normal. She had been married for 2 years and was yet to have a child. Initial FBC was Hb–7.7 g/dL; WBC–500.22 × 10^9^/L; platelet count–308 × 10^9^/L. Her ELTS score (1.9689) was consistent with intermediate‐risk disease. She was started on hydroxyurea until counts were low enough to have a BCR::ABL1 test done. The diagnosis was confirmed with BCR::ABL1 levels of 53.98%, and imatinib was started in March 2022. The patient presented with amenorrhea 5 months into therapy while in hematologic remission. Pregnancy was subsequently confirmed.The patient was counselled,referred to the antenatal clinic, and imatinib was discontinued. At 2 months of pregnancy, FBC findings were Hb–11.9 g/dL; WBC–4.7 × 10^9^/L; platelet count–154 × 10^9^/L; and BCR::ABL1 transcript level of 17.86%. She delivered at her primary care clinic and was subsequently lost to follow‐up by the Hematology Department. As a result, fetal outcomes and information on imatinib restart are not available.

Patient three is a 33‐year‐old female diagnosed in 2022. The patient had a 3‐month history of abdominal distention associated with night sweats and unintentional weight loss. Her spleen was palpable 8 cm below the left costal margin. Diagnosis was confirmed with BCR::ABL1 transcript levels of 52.49%. She delivered a live neonate via cesarean section seven months prior to presentation. Initial FBC was Hb–10.7 g/dL; WBC–142.69 × 10^9^/L; platelet count–769 × 10^9^/L. Her ELTS score (1.1549) was consistent with low‐risk disease. Treatment with imatinib 400 mg daily was initiated in October 2022, following the cessation of breastfeeding. Hematologic remission was achieved within two months of commencing imatinib. A follow‐up BCR::ABL1 test in June 2023 indicated a transcript level of 17.83%. In November 2024, the patient reported amenorrhea, and an ultrasound confirmed an intrauterine pregnancy at 5 weeks and 6 days gestation. Consequently, imatinib was withheld. Her FBC was Hb–9.8 g/dL; WBC–6.51 × 10^9^/L; platelet count–174 × 10^9^/L. A subsequent BCR::ABL1 test revealed a transcript level of 3.25%. She presented for 7 visits before delivering a live neonate (Table 2).

**TABLE 2:** FBC results of patient three during pregnancy.

	12/2024	01/2025	02/2025	03/2025	04/2025	05/2025	06/2025^∗^
HB (g/dL)	11.6	10.9	10.8	10.7	9.9	9.7	10.2
WBC (x 10^9^/L)	8.46	10.7	11.43	13.39	15.08	18.13	20.06
PLT (x 10^9^/L)	294	238	237	280	299	288	339

^∗^FBC done during the month of delivery.

The delivery was by spontaneous vaginal delivery. There were no adverse events or fetal abnormalities. The Apgar score at 1 min was 8/10 and 9/10 at 5 min. Results from the FBC done at the first visit after birth: Hb–11.7 g/dL; WBC–19.92 × 10^9^/L; platelet count–508 × 10^9^/L. Patient restarted imatinib in October 2025 and has currently attained a complete hematologic response.

An overview of the cases is presented in Table 3.

**TABLE 3:** Overview of key dates in the presented cases.

	Date of imatinib initiation	Confirmation of pregnancy and stop of imatinib	Date of delivery	Date of imatinib restart
Case #1	05/2015	05/2017	11/2017	04/2018
Case #2	03/2022	09/2022	05/2023	Lost to follow‐up
Case #3	10/2022	11/2024	06/2025	10/2025

## 3. Discussion

This study underscores a dimension of CML‐in‐pregnancy management that is sometimes underrepresented in the literature, especially in the sub‐Saharan African region, the decisive influence of cultural and social pressures on reproductive choices. In our setting, the desire to have children remains deeply valued, and people living with CML are not exempt from this expectation. As a result, pregnancy—planned or unplanned—must be anticipated and explicitly addressed from the moment of diagnosis [7].

Counselling at the stage of diagnosis is pivotal. There is an urgent need for structured education and frank conversations at the first visit, particularly in societies where cultural expectations are profound and therapeutic options during pregnancy are limited. Counselling should cover teratogenic risks of TKIs, reliable contraception, criteria and timing for safe treatment interruption, a contingency plan for unplanned pregnancy, and postpartum decisions about breastfeeding versus TKI resumption [8]. Documented, repeated counselling reduces decisional delays and aligns expectations among patients, families, and clinicians.

Preconception disease control is critical. The need to achieve and maintain molecular remission before attempting conception cannot be overemphasized in resource‐constrained environments. When interferon is unavailable and hydroxyurea is the main cytoreductive fallback, entering pregnancy in molecular remission widens the safety margin for pausing TKIs and lowers the likelihood of requiring cytoreduction during gestation. This is important because hydroxyurea may increase the risk of miscarriage and low birth weight [9]. These considerations should guide both contraception counselling and the timing of planned pregnancies.

Patient care should be envisioned as a shared journey. Successful outcomes require a partnership, where physicians and patients are co‐navigating choices with early and continuous involvement of the obstetric team. Joint hematology–obstetric reviews, agreed monitoring intervals (e.g., monthly FBCs), and predefined action thresholds improve clarity and reduce ad hoc decisions under pressure. This collaborative model respects patients’ reproductive goals while safeguarding maternal well‐being and fetal safety.

The 2025 European LeukemiaNet recommendations further support an individualized, pregnancy‐aware approach to CML care [10]. It emphasizes that TKIs should generally be avoided during pregnancy and stopped at pregnancy confirmation and acknowledges that selected women in the chronic phase with regular cycles and access to early pregnancy testing may continue TKI until the first positive pregnancy test. The recommendations also recognize watchful waiting as reasonable for patients with WBC below 100 × 10^9^/L, support interferon‐alpha as the preferred pregnancy‐compatible option for count control where available, and note that imatinib or nilotinib may be considered only after 16 weeks’ gestation in carefully selected situations. For clinical practice in resource‐limited settings, these recommendations reinforce the need for early counseling, rapid pregnancy detection, close hematology–obstetric collaboration, clear thresholds for intervention, and advocacy for access to interferon and molecular monitoring.

In terms of therapeutic options and access, where feasible, availability of alternatives to hydroxyurea—particularly interferon‐alpha—should be prioritized, as it expands options for women who cannot safely remain off therapy [11]. Even when hydroxyurea is used as second‐trimester cytoreduction, informed consent and affordability must be considered alongside drug supply, laboratory monitoring, and supportive care. Policies that secure consistent access and reduce out‐of‐pocket costs are integral to equitable care.

Importantly, one of the key challenges in resource‐limited settings is the restricted access to molecular monitoring. Current international recommendations emphasize serial BCR::ABL1 quantitative PCR testing to assess treatment response, guide treatment‐free remission strategies, and support clinical decision‐making [12]. However, in Ghana, molecular testing is largely financed through out‐of‐pocket payment and is therefore not universally accessible for regular monitoring. Consequently, clinicians often rely on clinical assessment and serial FBCs to monitor disease status during pregnancy, reserving molecular testing for situations where patients can afford the investigation or when a critical decision has to be made. This limitation is reflected in our case series, where regular molecular monitoring was not affordable for the patients. This highlights an important disparity between guideline‐recommended care and real‐world practice in many low‐ and middle‐income countries. Expanding access to affordable molecular diagnostics should therefore be considered a priority for improving the management of CML, particularly among women of reproductive age.

For centers like ours, a pragmatic framework emerges: (1) embed comprehensive counselling at diagnosis; (2) encourage planned conception only after sustained molecular remission; (3) stop TKIs upon pregnancy confirmation; (4) monitor with clear, communicated thresholds for intervention; (5) introduce cytoreduction after the first trimester only when clinically indicated and consent is given; and (6) align postpartum feeding plans with timely TKI re‐initiation. Parallel advocacy for the availability and affordability of interferon and other options will further strengthen this pathway.

In conclusion, in contexts where social pressure to bear children is strong and pregnancy‐safe treatments are limited, transparent counseling, a remission‐first strategy, and tightly coordinated hematology–obstetric care offer the best path to balancing reproductive autonomy with maternal and fetal safety.

## Author Contributions

Amma Benneh‐Akwasi Kuma, Leticia Lokko Hanson, and Nii Boi‐Doku Pepra‐Ameyaw collected the information on the cases. George Dennis Obeng and Marcell Csanádi provided support for the interpretation of the data.

## Funding

This study received no funding.

## Disclosure

All authors have read and approved the final version of the manuscript. Amma Benneh‐Akwasi Kuma had full access to all of the data in this study and takes complete responsibility for the integrity of the data and the accuracy of the data analysis.

## Consent

Informed consent was obtained from the patients verbally before the study and was documented in the patient’s medical records.

## Conflicts of Interest

The authors declare no conflicts of interest.

## Data Availability

The data that support the findings of this study are available from the corresponding author upon reasonable request.
